# Intact Female Mice Acquire Trace Eyeblink Conditioning Faster than Male and Ovariectomized Female Mice

**DOI:** 10.1523/ENEURO.0199-20.2021

**Published:** 2021-03-05

**Authors:** Amy P. Rapp, Craig Weiss, M. Matthew Oh, John F. Disterhoft

**Affiliations:** Department of Physiology, Feinberg School of Medicine, Northwestern University, Chicago, IL 60611

## Abstract

Female subjects have been widely excluded from past neuroscience work because of a number of biases, including the notion that cycling sex hormones increase variability. However, it is necessary to conduct behavioral research in mice that includes both sexes as mice are typically used for developing and evaluating future therapeutics. Understanding sex differences in learning is fundamental for the development of targeted therapies for numerous neurologic and neurodegenerative disorders, including Alzheimer’s disease, which is more prevalent in females than males. This study set out to confirm the role of sex and necessity of circulating ovarian hormones in the acquisition of the temporal associative memory task trace eyeblink conditioning (tEBC) in C57BL/6J mice. We present evidence that sex and ovarian hormones are important factors in learning. Specifically, intact female mice learn significantly faster than both male and ovariectomized (ovx) female mice. Data from pseudoconditioned control mice indicate that sex differences are because of differences in learned associations, not sensitization or spontaneous blink rate. This study strengthens the idea that ovarian hormones such as estrogen and progesterone significantly influence learning and memory and that further research is needed to determine the underlying mechanisms behind their effects. Overall, our findings emphasize the necessity of including both sexes in future behavioral studies.

## Significance Statement

Preclinical research commonly employs mice and it is imperative to understand differences between females and males that may impact the success of future therapies. Our study found that intact female mice learned at a faster rate than male and ovariectomized (ovx) female mice in trace eyeblink conditioning (tEBC), a temporal associative memory task. While all mice successfully acquired the task, ovx females were impaired compared with intact females throughout the course of training, including during the final day of training. These differences suggest additional research is needed on the role of ovarian hormones and the mechanisms underlying their effects on learning and memory.

## Introduction

Neuroscience research has largely neglected a fundamental variable, sex (Beery and Zucker, 2011; Shansky and Woolley, 2016). This bias has led to a disparity in knowledge of fundamental differences between males and females.

While many past behavioral experiments include a single sex, Shors and colleagues’ work in eyeblink conditioning (EBC) used both sexes, reporting that female rats outperformed male rats (Dalla and Shors, 2009). However, this finding has not been replicated in other species, including mice. It is necessary to assess sex differences in the mouse model, as mice are extensively used in preliminary clinical studies. Failure to include both sexes in the preclinical experiments that lay the foundation for future therapeutics may explain, in part, the differential effects between males and females observed in subsequent clinical trials (Soldin and Mattison, 2009). This is especially true for therapeutics aimed at treating neurologic and neurodegenerative disorders with known sex differences in severity and prevalence including schizophrenia, Alzheimer’s disease and anxiety (Zagni et al., 2016).

**Figure 1.**
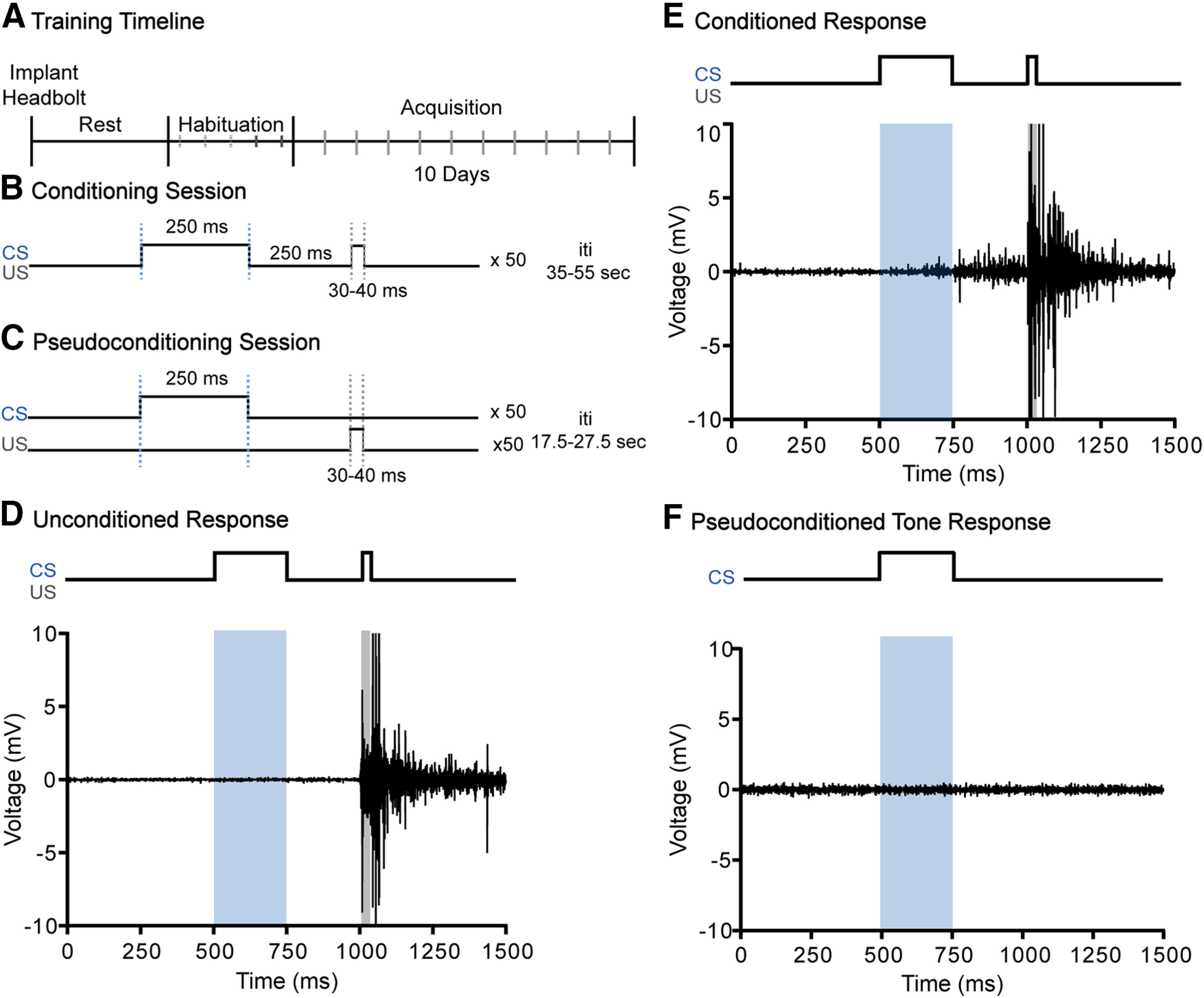
Experimental paradigm and example EMG traces. ***A***, Experimental timeline. ***B***, Conditioning session protocol. CS in blue, US in gray. ***C***, Pseudoconditioning session protocol. ***D***, EMG trace of unconditioned response to airpuff during early training. ***E***, EMG trace of CR to paired tone and airpuff during subsequent training sessions. ***F***, EMG trace of response to tone alone pseudoconditioning trial.

Therefore, we investigated acquisition of trace EBC (tEBC) in female and male C57BL/6J mice to determine the impact of sex in learning a hippocampal-dependent temporal associative memory task (Tseng et al., 2004). In this task, a neutral conditioned stimulus (CS) is paired with an aversive unconditioned stimulus (US), which causes a reflexive eyeblink response (Fig. 1*D*). The stimuli are separated by a stimulus free “trace” interval. Repeated presentation of the paired stimuli allows the subject to learn an association over time, leading to closure of the eye before the onset of the reflexive eyeblink, a conditioned response (CR; Fig. 1*E*). tEBC requires many trials to successfully acquire, allowing examination of the learning process and subsequent asymptotic performance. This task is also valuable because the circuitry of tEBC has been widely described across a number of species including rodents, rabbits and humans (McEchron and Disterhoft, 1997; Heiney et al., 2014; Weiss and Disterhoft, 2015; Lin et al., 2016).

Concerns about increased behavioral variability in female rodents because of circulating hormone levels during the estrous cycle have been widely expressed. However, recent studies suggest estrous cycle does not need to be monitored as females without a staged estrous cycle had similar variability as males in behavioral tasks (Prendergast et al., 2014; Fritz et al., 2017). To further investigate the necessity of circulating hormones for acquisition, we included an ovariectomized (ovx) female group in this study. We found intact female mice acquired tEBC significantly faster than male mice, however, the presence of circulating hormones was essential for their faster learning, as ovx females learned at a similar rate as males. All conditioned animals learned the associative learning task, reaching at least 60% adaptive CRs, although ovx female performance was impaired on the final day of training.

## Materials and Methods

### Animals

All procedures were approved by and completed in accordance with the Northwestern University Animal Care and Use Committee guidelines. Experiments were performed with young adult (three to four months) male, intact female, and ovx female C57BL/6J mice. All mice were obtained from The Jackson Laboratory. Ovariectomies were performed by The Jackson Laboratory at least two weeks before shipment. Estrous cycles of female mice were not monitored as previous studies have demonstrated females without a staged estrous cycle had similar variability as males in behavioral tasks (Prendergast et al., 2014). All mice were housed in Northwestern University temperature-controlled facilities in a 14/10 h light/dark cycle and fed *ad libitum*. Mice were group housed at arrival and allowed to acclimate to Northwestern University facilities for at least one week before experimentation. After headbolt implantation surgery, mice were housed individually.

### Surgery

Male (*n* = 29), intact female (*n* = 29), and ovx female (*n* = 24) mice were implanted with a custom headbolt two weeks before behavioral training. Animals were briefly anesthetized with 3–4% vaporized isoflurane mixed with oxygen (flow rate: 1–2 l/min). Buprenorphine (0.05–2 mg/kg) was administered subcutaneously as an analgesic. The scalp was shaved, and the mouse was placed in a stereotaxic device. The scalp was sterilized with iodine and 70% ethanol, then an incision was made along the midline. The skin was retracted laterally with microclips, and the skull was cleaned with 3% hydrogen peroxide then sterile saline. Two small stainless-steel screws (00–90) were implanted to the left of midline (one in front of bregma, and one in front of lambda). The bare stainless steel groundwire (0.005 inches; AM Systems: 792800) of the custom headbolt was wrapped around the screws in a figure-eight pattern to serve as a ground for electromyogram (EMG) recordings. A thin layer of Metabond adhesive cement (Parkell) was spread over the skull, screws, and wire to secure them in place. To expose the muscle and place EMG wires, the skin surrounding the right eye was retracted. Four polyimide-coated stainless steel (0.005 inches; PlasticsOne: 005 sw/2.0 37365 SS) wires with 2–3 mm of exposed wire were placed on the muscularis orbicularis oculi for EMG recording. The headbolt piece and base of the EMG recording wires were then secured with additional adhesive cement. The skin was released from microclips and placed over the cement. Skin was allowed to rest naturally, and the exposed area was sealed with additional adhesive cement. Animals recovered on a warm heating pad before being returned to their home cage. Animals were allowed 5–7 d to recover before habituation began.

### tEBC

**Figure 2.**
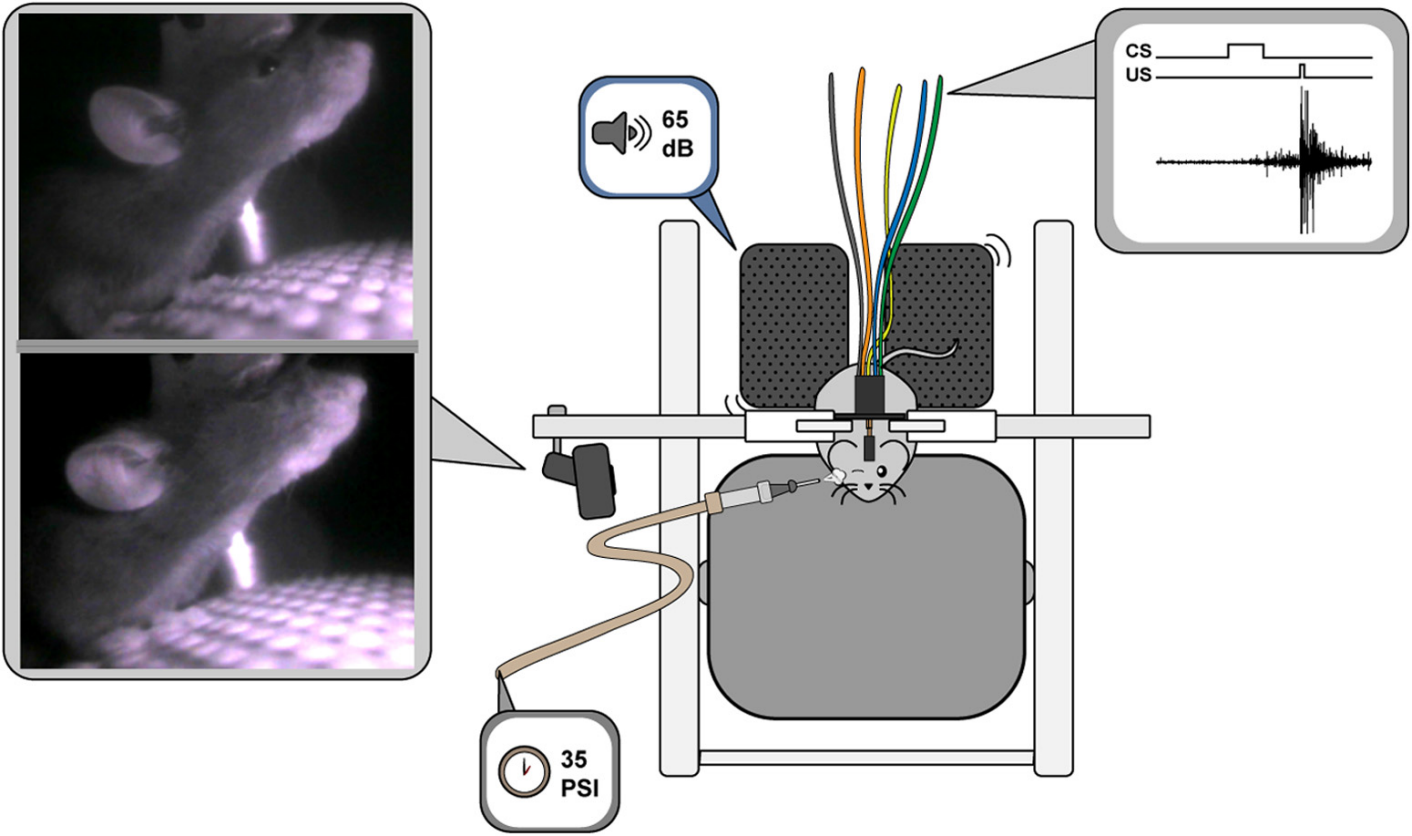
Schematic of EBC behavioral apparatus. Upper left. Video display of mouse with open eye during baseline. Lower left. Display depicting mouse with closed eyelids during a CR. Middle. Depiction of head-fixed mouse atop the freely rotating cylinder. Camera for visualizing mouse during conditioning task on left of cylinder frame. Speakers behind mouse are used to deliver tone CS. Blunted needle delivers aversive airpuff US to eye. Custom headbolt implanted on the mouse’s head connects to amplifier to receive EMG signal (depicted on the right).

Before behavioral training, mice were handled for 3 d for 5 min/d to habituate mice to restraint and the experimenter. After 3 d of handling, mice were habituated to head-fixation on a moveable cylinder apparatus for the length of a training session without the presentation of stimuli. Training began 2 d following habituation. Training consisted of one session per day for 10 d (Fig. 1*A*). Mice were randomly assigned to either a conditioned group or pseudoconditioned group. Conditioned animals received a 65 ± 2 dB tone (250 ms, 2 kHz) CS paired with a 35 ± 5 PSI corneal airpuff (30–40 ms) US (Fig. 1*B*). Each conditioning session consisted of 50 paired CS/US trials with a random 35- to 55-s intertrial interval. Pseudoconditioned animals received 50 CS-alone trials and 50 US-alone trials in pseudorandomized order with a 17.5- to 27.5-s intertrial interval (Fig. 2*C*,*F*). Custom routines in LabVIEW (National Instruments) were used for stimulus presentation procedures, data collection, storage, and analysis (routines available on request). Tone intensity was calibrated with a sound meter, placed where the mouse would be, at the start of each day of training. Air pressure was calibrated with a manometer (Thermo Fisher Scientific) secured at the output of a 0.5-inch 16-gauge blunted needle before each training session. Animals were visually monitored during training through a camera (Logitech C270) attached to the frame of the cylinder apparatus (Fig. 2). Trials were not presented when the animal was visibly moving.

### Data analysis

EMG signal output was amplified (5000×) and filtered (100 Hz to 5 kHz), then digitized at 3 kHz and stored by computer. For analysis, EMG data were rectified and integrated with a 10-ms time constant.

A CR was defined as increased EMG activity lasting at least 15 ms with an amplitude at least four SDs above the mean baseline activity. Baseline activity was the average EMG activity starting 250 ms before CS onset (see Fig. 1*E*). Trials were excluded if baseline activity was 2 SDs above the mean baseline activity for the session. CR onset was calculated in reference to the start of the tone CS. An adaptive CR was defined as a CR that was present in the 200 ms before US onset. Animals that reached at least 60% adaptive CRs were considered to have learned the task. The number of trials to eight consecutive CRs was also used as a measurement of learning.

Data were analyzed with Bartlett’s test, two-way repeated measures ANOVA or mixed-effects analysis, one-way ANOVA, and *post hoc* Šídák’s multiple comparisons test or Tukey’s multiple comparison test, when appropriate (Prism v8; Table 1). The probability level of *p* ≤ 0.05 was used as an indicator of statistical significance. Data are expressed with Standard Error of the Mean (SEM). Statistical tests did not include data from habituation, except for direct habituation comparison. Mice were excluded from analysis because of poor health, high startle response or failure to learn delay conditioning (intact female *n* = 6; male *n* = 4; ovx *n* = 5). High startle response was defined as activity occurring during the 50 ms after the start of the CS which is greater than average activity + 4 SD on two or more sessions. Delay conditioning is non-hippocampal dependent, where the stimuli overlap (the length of the CS is extended, and the US and CS co-terminate). Failure to learn delay conditioning indicates a possible brainstem/cerebellar deficit (Cheng et al., 2008; Heiney et al., 2014; Yang et al., 2015).

**Table 1:** Statistical table

Figure		Test	*F* value	*p* value	Standard Omega-Square	*R*^2^	Mean difference
3	Habituation	Two-way repeated measures ANOVA	2,64				
		Sex	0.01855	0.9816	0.03724		
	Variance	Ordinary one-way ANOVA Bartlett’s test	Bartlett’s statistic				
		Trained intact Female, male, ovx	0.8188	0.6641			
	Pseudo	Repeated measures ANOVA	2,14; 9,126; 18,126				
		Sex	0.6797	0.5227	4.700		
		Session	0.9406	0.3991	2.801		
		Interaction (session * sex)	0.4007	0.9857	2.387		
	Conditioned vs pseudo	Two-way repeated measures ANOVA	1,65; 9,585; 9,585; 65,585				
		Group	18.55	<0.0001	10.25		
		Session	21.74	<0.0001	8.267		
		Interaction (session * group)	18.22	<0.0001	6.926		
		Subject	13.07	<0.0001	35.89		
		Šídák’s multiple comparisons test					
		T5		0.141			19.72
		T6		0.0899			20.57
		T7		0.0001			31.650
		T8		<0.0001			40.820
		T9		<0.0001			42.14
		T10		<0.0001			46.69
	Sex difference	Two-way repeated measures ANOVA	2,47; 9,423; 18,423; 47,423				
		Sex	4.447	0.017	5.962		
		Session	76.07	<0.0001	37.09		
		Interaction (session * sex)	1.471	0.0961	1.434		
		Subject	12.37	<0.0001	31.51		
		Tukey’s multiple comparisons test					Predicted (LS) mean
							difference
		Intact female, male		0.0421			14.04
		Intact female, ovx		0.0318			15.72
		Male, ovx		0.9581			1.678
		Tukey’s multiple comparisons test					
		T2					Predicted (LS) mean difference
		Intact female, male		0.0086			22.04
		Intact female, ovx		0.0352			19.68
		Male, ovx		0.9524			−2.353
		T3					
		Intact female, Male		0.0088			21.98
		Intact female, ovx		0.0266			20.5
		Male, ovx		0.9808			−1.484
		T4					
		Intact female, male		0.004			23.78
		Intact female, ovx		0.0356			19.65
		Male, ovx		0.8605			−4.133
		T5					
		Intact female, male		0.0247			19.38
		Intact female, ovx		0.3594			10.81
		Male, ovx		0.525			−8.57
		T10					
		Intact female, male		0.4758			8.609
		Intact female, ovx		0.0167			21.81
		Male, ovx		0.2186			13.2
4	8 Consecutive CRs	Ordinary one-way ANOVA	2,47				
		Sex	4.651	0.0144		0.1652	
		Tukey’s multiple comparisons test					
		Intact female, male		0.0485			−107.9
		Intact female, ovx		0.0218			−131.1
		Male, ovx		0.8775			−23.13
5	CR onset	Mixed-effects analysis	9,422; 2,47;18,422				
		Session	2.770	0.0433			
		Sex	2.978	0.0606			
		Interaction (session * sex)	0.5707	0.9202			
		Tukey’s multiple comparisons test					
		T9					
		Intact female, male		0.1464			−31.63
		Intact female, ovx		0.0210			−34.21
		Male, ovx		0.9858			−2.580
		Ordinary one-way ANOVA	2,27				
		Sex	15.40	<0.0001		0.5035	
		Tukey’s multiple comparisons test					
		Intact female, Male		<0.0001			−25.42
		Intact female, ovx		0.0011			−19.28
		Male, ovx		0.4167			6.133

## Results

**Figure 3.**
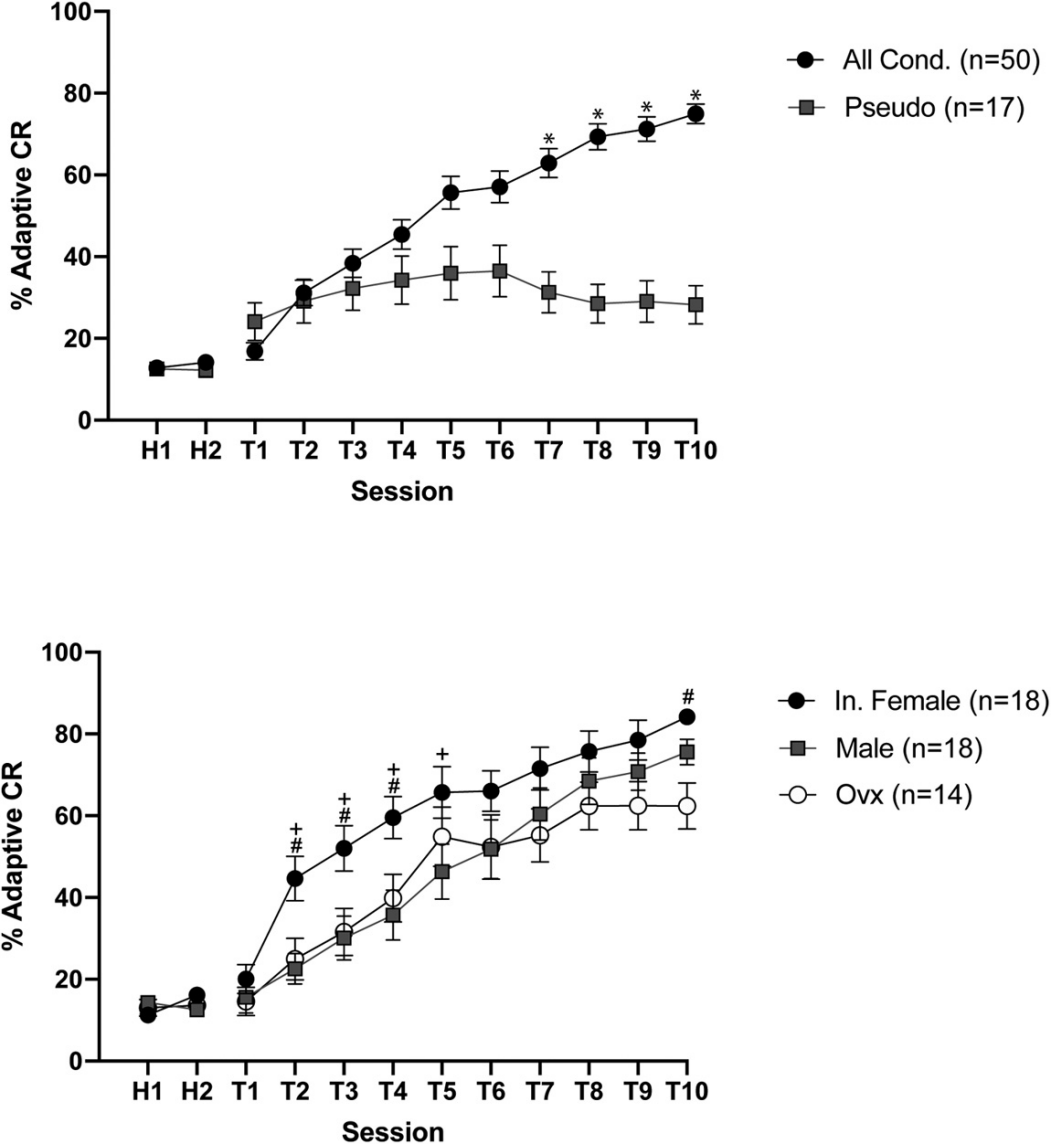
Upper. Percentage adaptive CRs for all conditioned animals (males, intact females, ovx) and pseudoconditioned animals across 2 d of habituation and 10 d of training. Mean ± SEM shown. * Šídák’s multiple comparisons test *p* < 0.05. Lower. Percentage adaptive CRs for intact females, male, and ovx female animals. Intact females learn significantly faster than males and ovx females. + Tukey’s multiple comparison test *p* < 0.05 intact female versus male; # Tukey’s multiple comparison test *p* < 0.05 intact female versus ovx.

All groups of mice displayed similar low levels of spontaneous blinking during habituation (*F*_(2,64)_ = 0.01855, *p* = 0.9816). Pseudoconditioned mice (male, intact female, ovx) responded comparably throughout the 10 training sessions (*F*_(2,14)_ = 0.6797 *p* = 0.5227) and were grouped together for analysis. All conditioned mice (intact female, male, ovx) reached learning criterion (60% adaptive CRs), in contrast to the pseudoconditioned controls. As shown in Figure 3, a two-way repeated measures ANOVA of % adaptive CRs revealed a significant increase in adaptive CRs for the conditioned mice compared with pseudoconditioned mice (*F*_(1,65)_ = 18.55, *p* < 0.0001).

While male, intact female, and ovx mice trained on tEBC acquired the task over the course of 10 training sessions (*F*_(9,423)_ = 76.07, *p* < 0.0001), a significant difference between the sexes was observed (*F*_(2,47)_ = 4.447, *p* = 0.0170). Conditioned intact female mice learned significantly faster than ovx females and males (*F*_(2,47)_ = 4.447, *p* = 0.0170: Tukey’s multiple comparison test intact female vs male, *p* = 0.0421, intact female vs ovx, *p* = 0.0318, male vs ovx *p* = 0.9581; Fig. 3). Planned comparisons of sessions 2–5 (where initial acquisition is occurring) indicated that intact females exhibited a significantly greater percentage of adaptive CRs relative to male and ovx mice [*F*_(2,47)_ = 4.447, *p* = 0.0170: Tukey’s multiple comparison tests, T2, intact female vs male (*p* = 0.0086), intact female vs ovx (*p* = 0.0352), T3, intact female vs male (*p* = 0.0088) intact female vs ovx (*p* = 0.0266), T4, intact female vs male (*p* = 0.004) intact female vs ovx (*p* = 0.0356), T5, intact female vs male (*p* = 0.0247)].

All intact female, male, and ovx female mice reached at least 60% adaptive CR by the last day of training. However, ovx females were impaired during the final day of training compared with intact females (T10, Tukey’s multiple comparison test, *p* = 0.0167).

The variance of intact females, males, and ovx females was not significantly different on the first day of training [Bartlett’s test (corrected) = 0.8188, *p* = 0.6641]. This further supports the findings of previous studies that variability in behavioral tasks are similar between males and females without a staged estrous cycle (Prendergast et al., 2014; Fritz et al., 2017).

**Figure 4.**
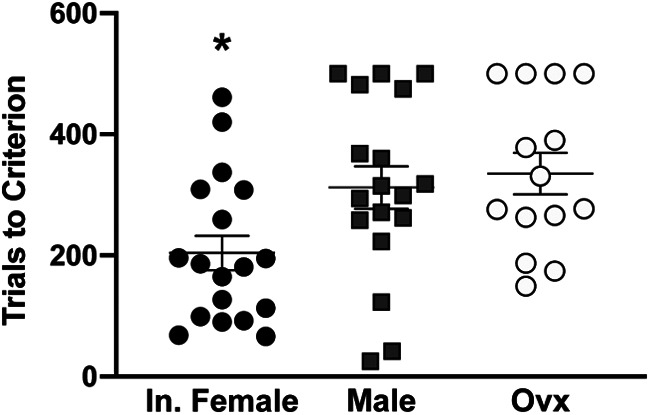
Intact females learn significantly faster than ovx females and males. Intact females reach eight consecutive CRs in 204 trials while males and ovx require 311 and 335 trials, respectively. Mean ± SEM shown; * Tukey’s multiple comparison test *p* < 0.05 for intact female versus male and ovx.

Number of trials to consecutive eight CRs was also used as a measurement of learning rate. Animals that failed to reach eight consecutive CRs by the end of 10 training sessions were scored as 500 trials, the total number of conditioning trials (males *n* = 3, ovx *n* = 4). Intact females reached eight consecutive CRs significantly faster than both males and ovx females (Tukey’s multiple comparison test, intact female vs male, *p* = 0.0485, intact female vs ovx, *p* = 0.0218). On average, intact females reached eight consecutive CRs in 204 trials, while ovx females required 335.1 trials and males required 311.9 trials (Fig. 4).

**Figure 5.**
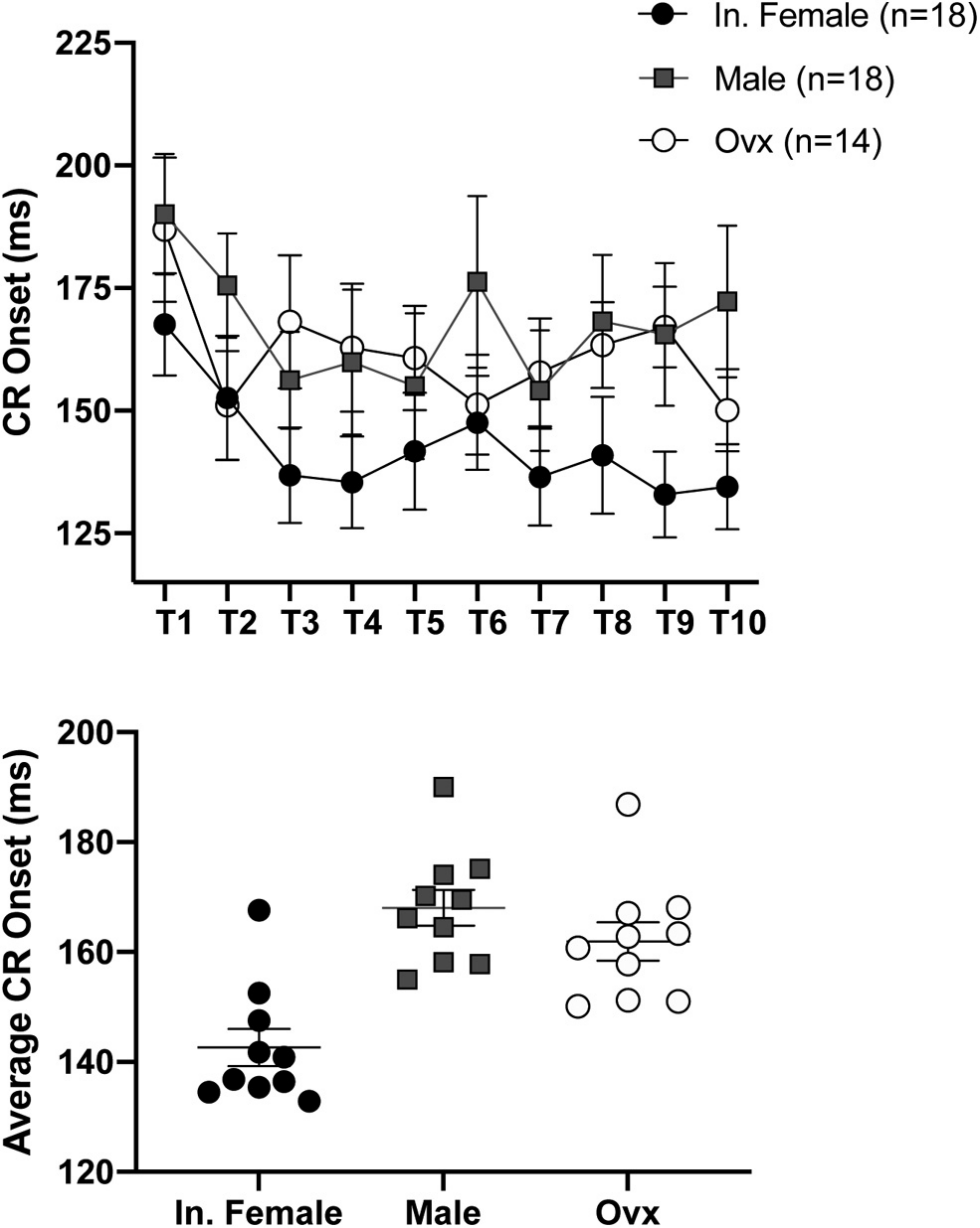
Average CR onset latency. Mean ± SEM shown. Upper. Intact females respond earlier in the trial on average across all sessions. Lower. Average CR onset latency across all training sessions. Intact female *n* = 18, male *n* = 18, ovx *n* = 14, per training session.

Analyses of the CR onset latency revealed a significant effect of training sessions (*F*_(9,422)_ = 2.770, *p* = 0.0433) and a trend for a difference among the groups (mixed-effects analysis, *F*_(2,47)_ = 2.978, *p* = 0.0606; Fig. 5). Across all days of conditioning, intact females’ average response onset latency was 142.6 ms after the start of the CS, while males and ovx females responded at 167.3 and 161.9 ms, respectively (Fig. 5).

## Discussion

This study set out to confirm and extend the differences in acquisition of a temporal associative memory task, tEBC, because of sex and circulating ovarian hormones in mice. We found that intact female mice learned tEBC at a faster rate compared with males and ovx mice. Intact females also exhibited earlier CR onset times. These results indicate that the intact female mice learn to anticipate the aversive stimulus more quickly and respond more rapidly during the trial than males or ovx mice. These findings parallel those observed by Shors and colleagues, who found female rats learned tEBC faster than male rats (Shors et al., 1998; Wood and Shors, 1998; Dalla et al., 2009). It is important to note that these sex differences were not only confirmed across species but across distinct experimental parameters. While both studies used tEBC, the experiments varied in a number of technical ways including: the length of the trace period, the modality of aversive US, and number of trials delivered each day. Additionally, our study directly compared intact female, male, and ovx female mice. Our results highlight the impact that sex has on learning is not dependent on specific experimental protocols.

There have been conflicting reports of the effects of ovariectomy on different forms of memory including spatial memory and object recognition. Ovariectomy has been shown to impair spatial working memory in radial arm maze and non-spatial memory in object recognition (Daniel et al., 1997; Wallace et al., 2006). However, other studies have shown ovariectomy can improve or have no effect on spatial memory in Morris water maze (Singh et al., 1994; Bimonte-Nelson et al., 2003). Previous tEBC studies demonstrated that the removal of ovarian hormones eliminates stress-induced sex differences and decreased performance late in training (Wood and Shors, 1998). Similarly, our present findings reveal that ovariectomy slows the learning of a temporal associative memory task in females and reduces performance on the final day of conditioning. This decline in learning may be because of decreased spine density in CA1 and medial prefrontal cortex pyramidal cells because of ovariectomy (Gould et al., 1990; Wallace et al., 2006). However, the ovx females in our study still successfully acquired the task and reached the learning criterion of 60% adaptive CRs.

Our findings suggest that cycling ovarian hormones are necessary for the enhanced learning rate in females. Enhancement of associative learning because of ovarian hormones, such as estrogen, is in line with evidence that estrogen plays a functional role in learning and memory formation. It is known that exposure to estrogen in young female rodents increases density of dendritic spines in CA1, neurogenesis in dentate gyrus and synaptic plasticity (Gould et al., 1990; Woolley and McEwen, 1993; Smith et al., 2009; Frick et al., 2018). These mechanisms may mediate intact females’ enhancement in acquisition of this hippocampal-dependent associative memory task. In ovx female rats, increased levels of estrogen have been shown to enhance acquisition of tEBC as compared with vehicle treated rats, albeit at supraphysiological doses (Leuner et al., 2004). Additionally, it was reported that female rats acquire tEBC at a faster rate when in proestrous (high estrogen levels), compared with rats at either estrus or diestrus (Shors et al., 1998). However, there is rapid fluctuation of ovarian hormones within each phase of the estrous cycle (Frick et al., 2015). During the proestrus phase, in particular, hormone levels rise during the day and peak in the evening. Therefore, it is difficult to assess learning and memory within a single phase of the cycle (Frick et al., 2015), especially for tEBC which requires several daily training sessions to acquire. While we did not measure the estrous cycle, we found that intact female mice learned tEBC at a faster rate than male and ovx female mice in the absence of staged estrous cycle. Moreover, the variability of the intact female mice was not significantly different from that of male and ovx mice, further supporting a previous report that intact females without staged estrous cycle had similar variability as males (Prendergast et al., 2014).

Estrogens may also play a role in male mice during acquisition of tEBC. Testosterone is aromatized to estradiol in the central nervous system and has been shown to enhance cognition in humans and animals (Edinger and Frye, 2007; Luine, 2014). Estrogen receptor agonists increase CA1 spine density *in vivo* and *in vitro* in males, indicating that estrogen may also influence learning in males (Murakami et al., 2006, 2015; Jacome et al., 2016; Koss and Frick, 2017). Gonadectomy has also been shown to impair male performance on a variety of tasks (Frye et al., 2004), including object recognition (Ceccarelli et al., 2001), T-maze (Kritzer et al., 2001), and radial arm maze (Harrell et al., 1990).

Our results confirm and extend the findings that intact females learn significantly faster than both ovx females and males on the tEBC task. Although all conditioned animals acquired the task, ovx females’ performance was impaired compared with that of intact females on the final day of training. These differences in learned responses cannot be attributed to sensitization to stimuli or differences in spontaneous blink rate, since no difference was observed in spontaneous blink rate or response to the tone among the female, male, and ovx female pseudoconditioned controls. Overall, these results emphasize the need for inclusion of both females and males in behavioral neuroscience studies. Behavioral tasks are used as the benchmark for clinical drug studies for neurologic and neurodegenerative disorders. If sex is not factored in as a biological variable, critical differences essential to successful treatments may go undetected.

## Synthesis

Reviewing Editor: Karyn Frick, University of Wisconsin-Milwaukee Decisions are customarily a result of the Reviewing Editor and the peer reviewers coming together and discussing their recommendations until a consensus is reached. When revisions are invited, a fact-based synthesis statement explaining their decision and outlining what is needed to prepare a revision will be listed below. The following reviewer(s) agreed to reveal their identity: Natalie Tronson, Thomas Foster.

The authors have sufficiently addressed the reviewer's concerns.
